# Stress Relaxation Behavior of Poly(Methyl Methacrylate)/Graphene Composites: Ultraviolet Irradiation

**DOI:** 10.3390/polym14194192

**Published:** 2022-10-06

**Authors:** Yu-Cheng Ju, Donyau Chiang, Ming-Yen Tsai, Hao Ouyang, Sanboh Lee

**Affiliations:** 1 Department of Materials Science and Engineering, National Tsing Hua University, Hsinchu 300, Taiwan; 2 National Applied Research Laboratories, Taiwan Instrument Research Institute, Hsinchu 300, Taiwan

**Keywords:** poly(methyl methacrylate), graphene, stress relaxation, Kelvin representation of linear standard solid model, activation energy

## Abstract

The graphene/poly (methyl methacrylate) (PMMA) composites are a promising candidate for electronic, optoelectrical, and environmental applications. Understanding the mechanical degradation of PMMA-based materials is of practical importance in improving the reliability and lifespan of the associated structures and systems. In this study, we investigate the effects of functionalized graphene (FG) and UV irradiation on the stress–relaxation of PMMA. Uniaxial tensile and stress –relaxation tests are performed to evaluate the mechanical properties of the composites. The mechanical strength and elongation at the break increase with the graphene concentration but decrease with the increase of the irradiation dose. Raman spectroscopy and intrinsic viscosity measurement are applied to examine the root cause of the degradation in the composites. UV irradiation leads to polymer chain scission and loss of molecular weight. The Kelvin representation of the standard linear solid model (SLSM) is used to describe the stress–relaxation curves of the composites. The value of the elastic modulus in the Kelvin element decreases with the increase in temperature. The viscosity follows the Arrhenius equation. The activation energy of viscosity increases with the increasing FGs concentration because the FGs hinder the chain motion of PMMA. However, UV irradiation makes chain scission of PMMA/FGs composite so that the polymer chain moves more easily and the activation energy of stress relaxation lowers. The steady-state stress follows the van ’t Hoff equation that stress relaxation is an exothermal deformation process. Although Maxwell’s representation of SLSM is mathematically identical to the Kelvin representation of SLSM, the former cannot interpret the stress–relaxation behavior of PMMA/FGs composite, which is against the concept of Young’s modulus as a decreasing temperature function.

## 1. Introduction

Poly(methyl methacrylate) (PMMA) is a thermoplastic and versatile polymer. PMMA possesses good mechanical properties, fabricability, and biocompatibility, which means it can be used in a variety of applications, such as in optical, biomedical, and communication industries [1,2,3,4]. The methyl group (CH_3_) is a pendant group of PMMA and prevents the polymer chain from intimating close to forming a crystalline structure, which causes PMMA to be amorphous and results in transparency and low density. The pendant group blocks the polymer chain slip and leads to PMMA being brittle [5]. Due to its biocompatibility, PMMA is regarded as an ideal organic glass material and is manufactured to be an artificial intraocular lens to substitute for damaged human lenses [6].

The brittleness and low-glass transition temperature of PMMA limits its applications. Three methods have been extensively investigated to modify the structure and improve PMMA properties. There are comonomer, additives, and filler mixing. In the comonomer strategy, a small number of comonomers are added and polymerized with methyl methacrylate (MMA) monomers to synthesize the copolymer. Gupta et al. prepared MMA and methacrylic acid monomers in different ratios and produced copolymers [7]. The addition of methacrylic acid up to 25 wt% reduced the microbial adhesion without significantly affecting the flexural strength. For the second strategy, additives such as stabilizers and plasticizers are added to polymers to change their properties. Street et al. [8] mixed MMA with self-dimerizing hydrogen bond monomer, UPyMA, to polymerize the p(MMA-r-UPyMA) copolymer. The additives of UPyMA into MMA can overcome the mechanical limitation for 3D printed material without affecting melt processability. For the last strategy, filler addition is the most popular approach to improve PMMA performance. Chew et al. [9] blended ceramic fillers, SiO_2_ and Al_2_O_3_, into PMMA-based polymer electrolytes to increase the number of charge carriers, and enhance electrolyte conduction. Sun et al. [10] conducted the solution mixing between colloidal zinc oxide quantum dots of a uniform 5 nm particle size and PMMA matrix. The PMMA/ZnO composite films showed high UV shielding efficiency and good transparency. Avella et al. [11] synthesized PMMA/CaCO_3_ composites using in-situ polymerization to improve the mechanical properties, in which the abrasive resistance of the composite was increased due to the fine dispersion of the nanoparticles.

Graphene possesses extraordinary mechanical properties, good thermal conductivity, and electrical conductivity due to its unique carbon atom arrangement [12,13,14]. Those remarkable properties promote graphene as an ideal filler to enhance the polymer composite performance. However, the strong van der Waals force between the nanosheet layers causes graphene agglomeration and results in poor dispersion [15]. Several studies added a surfactant or functionalized graphene nanosheet [16]. They were investigated and focused on the graphene dispersion in the polymer matrix. Wan et al. used Triton X-100 to improve the graphene dispersion in an epoxy matrix and enhanced the thermal stability of epoxy/graphene composites [17]. Goncalves et al. [18] synthesized PMMA/Graphene Oxide (GO) composites via atom transfer radical polymerization and improved the mechanical properties, including Young’s modulus, elongation at break, and tensile strength with 1 wt% GO addition. Ramanathan et al. [19] manufactured PMMA/graphene composites using the solution mixing method and found the glass transition temperature had a 30 °C increase and elastic modulus a 33% increase by adding only 0.01 wt% FGs. To avoid graphene agglomeration, there is an alternative strategy based on the polymerization occurring in the interlayer spaces of graphite or graphite intercalation compounds [20,21,22]. 

When the polymers are irradiated by ultraviolet (UV) light, polymer degradation happens. UV irradiation leads to photodegradation and results in polymer chain scission and molecular weight reduction [23]. The mechanical properties of polymers, such as fracture stress, elongation at break, and Young’s modulus, are weakened after UV exposure and the polymers become brittle [24,25]. PMMA irradiated by UV with wavelengths of 193 nm, 248 nm, and 308 nm and different doses were investigated by Wochnowski et al. [26]. The crosslinking reaction of the ester side chain between two PMMA molecular chains led to the UV-curing process in wavelengths below 250 nm and low UV dose. The side chain was cleaved from the polymer main chain at medium doses. The chain scission occurred at a high dose and resulted in the polymer structure defragmentation. 

Studies of the mechanical behavior of polymeric composites have included the fracture, tensile strength, hardness, deformation, and stability of various forms. This paper focused on stress–relaxation behavior. The variation of stress with time is monitored when the materials are subjected to constant strain at different temperatures. Many models were proposed to interpret the viscoelastic behavior of the polymers [27,28,29,30,31,32,33,34,35,36]. Kohlrausch–Williams–Watts function is an empirical formula and is most popularly used to describe stress–relaxation behavior [27,28,29]. The standard linear solid model (SLSM) is an alternative to model stress–relaxation behavior [30,31,32,33]. Ariyama et al. fitted the stress–relaxation data of polypropylene by Kelvin representation of SLSM and calculated the activation energy of 210 kJ/mol for a stress–relaxation process [33]. Several scholars tried to add elements to the model to best fit the stress–relaxation data [34,35]. Vaidyanathan performed the time–temperature superposition principle to predict long-term stress–relaxation behavior from the short-term data [36]. 

The PMMA/graphene composites are a promising candidate in electronic, optoelectrical, and environmental applications. Understanding the mechanical degradation of PMMA-based materials is of practical importance in improving the reliability and lifespan of the associated structures and systems. In this study, we investigate the effects of functionalized graphene (FG) and UV irradiation on the stress relaxation of PMMA. Raman spectroscopy and intrinsic viscosity measurements are conducted to examine the FGs’ roles in the strength measurement and the UV photodegradation mechanism. Uniaxial tensile and stress–relaxation tests are employed to evaluate the mechanical properties of the PMMA/FGs composites. The Kelvin representation of SLSM is used to interpret the stress–relaxation data. 

## 2. Kelvin Representation of the Standard Linear Solid Model

**Figure 1:**
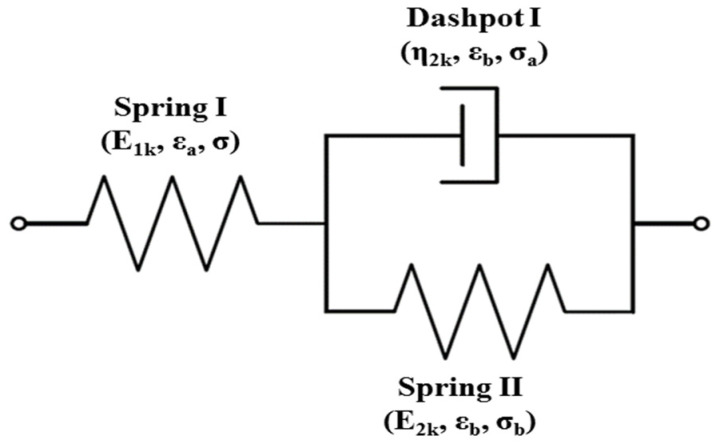
Schematic of standard linear solid model for stress relaxation: Kelvin representation.

The Kelvin representation of the standard linear solid model (SLSM), as shown in Figure 1, is applied to simulate the stress–relaxation behavior of polymeric composite. The spring I and a Kelvin element, which consists of a spring II and a dashpot I connected in parallel, are connected in series for the Kelvin representation. We consider the spring I under the action of stress σ

(1) $$\varepsilon_{a}=\sigma /E_{1k}$$

The applied stress on the Kelvin element is the sum of the stress in spring II (*σ*_b_) and the stress in dashpot I (*σ*_a_).

(2) $$\sigma =\sigma_{a}+\sigma_{b}=\eta_{2k}\frac{d\varepsilon_{b}}{dt}+E_{2k}\varepsilon_{b}$$

The total strain can be expressed as

(3) $$\varepsilon =\varepsilon_{a}+\varepsilon_{b}$$

Substituting Equation (3) into Equation (2) yields Equation (4),

(4) $$\sigma =\eta_{2k}\frac{d\left(\varepsilon -\varepsilon_{a}\right)}{dt}+E_{2k}\left(\varepsilon -\varepsilon_{a}\right)$$

Assume that the total strain is maintained constant, *dε*/*dt* = 0, during the stress–relaxation process. Combining Equation (1) and Equation (4), the first-order linear differential equation is obtained as

(5) $$\frac{d\sigma }{dt}\left(\frac{E_{1k}+E_{2k}}{\eta_{2k}}\right)\sigma =\frac{E_{1k}E_{2k}}{\eta_{2k}}\varepsilon$$

At the initial time, only spring I experiences a stress $\sigma (=E_{1k}\varepsilon$). Solving Equation (5) with the initial time of $\sigma =E_{1k}\varepsilon$ yields the solution for Kelvin representation of SLSM as

(6) $$\sigma \left(t\right)=\frac{E_{1k}E_{2k}\varepsilon }{E_{1k}+E_{k2}}+\frac{E_{1k}{}^{2}\varepsilon }{E_{1k}+E_{2k}}exp\;\left[-\frac{\left(E_{1k}+E_{2k}\right)t}{\eta_{2k}}\right]=\frac{E_{1k}E_{2k}\varepsilon }{E_{1k}+E_{2k}}+\frac{E_{1k}{}^{2}\varepsilon }{E_{1k}+E_{2k}}exp\;\left[-\beta_{s}t\right]\;$$

Equation (6) is used to fit the stress–relaxation data of the PMMA/FGs composite. Note that *β*_s_ = (*E*_1*k*_ + *E*_2*k*_)/*η*_2*k*_. Note that the Maxwell representation of the standard linear solid model, as shown in Figure S1, is analyzed in Supplementary Information.

## 3. Experimental

The methyl methacrylate (MMA) monomers and functionalized graphene sheets (FG) with the carboxyl functional group were purchased from Sigma–Aldrich Co. (St. Louis, MO, USA) and Euflex Technology Co. (New Taipei City, Taiwan), respectively. The radical initiator of 2,2-azobisisobutyronitrile (AIBN) was obtained from Aencore Chemical Co., Ltd. (Victoria, Australia). The physical data for graphene nanosheets are bulk density ~0.215 g/cm^3^, specific surface area ~20 m^2^/g, flake planar size 0.3~5 µm, flake thickness < 50 nm, and no detectable amorphous carbon.

The PMMA/FGs composites with FGs of 0, 0.3, and 0.7 wt% concentrations were prepared in-house. MMA monomers of 40 mL (38 g) mixed with different concentrations of FGs were prepared in glass flasks, covered with aluminum foil, and ultra-sonicated in a water bath of 50 °C for 1.5 h. Then, 0.5 wt% concentration of AIBN was gradually added to the mixed solution and continued to ultra-sonicate for another 1.5 h. After the ultrasonication, the solution was poured into another glass flask, sealed with aluminum foil, and immersed in a water bath of 80 °C for 20~25 min. During this process, called the pre-polymerization step of MMA monomers, the viscosity of the solution gradually became stickier during the heating. Then, we put the viscous solution in a template and immersed it in another water bath of 60 °C for 24 h to ensure a fully complete polymerization reaction. Finally, the as-received composites were dried in a vacuum oven at 90 °C for 24 h to evaporate the residual MMA monomers.

The PMMA/FGs composites were cut into a dumbbell shape by laser. The thicknesses of the dumbbell specimens are 1.5 mm and 0.8 mm for the tensile test and stress–relaxation test, respectively. The specimens were ground with 180, 800, 1200, and 2500 grit cabinet papers and polished with 1 µm alumina slurry. After polishing, all specimens were annealed at 90 °C in the air for a day and furnace-cooled to ambient temperature to release the residual stress induced by the preparation processes.

The ultraviolet (UV) irradiation system (Kingo Electric Co., Tainan, Taiwan) is a drawer-type curing machine with a 1 kW high-pressure vapor mercury lamp. Two filters were inserted between the UV source and specimen. One filter cut the UV wavelength greater than 254 nm and the other blocked the IR radiation to maintain the temperature of 38 °C in the sample chamber. The specimens were exposed at the dose rate of 1.57 mW/cm^2^ of UV light for 2 h and 4 h and the total doses were equivalent to 11.3 and 22.6 J/cm^2^, respectively.

The PMMA/FGs specimens with FGs of different concentrations and UV doses were used. The specimens with a thickness of 1.5 mm were conducted during a tensile test at room temperature using the tensile test machine (model PT-1699V, Perfect International Instrument Co. Ltd., Taichung, Taiwan) with a crosshead speed of 10 mm/min. All data were averaged from three specimens under the same condition. The stress–relaxation test was operated with the dynamic mechanical analyzer (TA Q800, TA Instrument Co., New Castle, DE, USA). The specimen has a thickness of 0.8 mm. The strain of 0.5% and temperatures ranging from 50 °C to 80 °C were employed. Each specimen was maintained at the predetermined temperature for 2 min before the stress –relaxation test started to ensure the specimen temperature reaches the thermal equilibrium state.

Intrinsic viscosity measurement was undergone at 25 °C with the glass viscometer (Cannon-Ubbelohde 6983S, State College, PA, USA). The solvent to dissolve the PMMX/FGs composites was tetrahydrofuran (THF) with concentrations of 0.05, 0.1 0.15, and 0.2 g/dL in the viscosity measurement. Three data were averaged to decide the efflux time for each concentration. Raman spectroscopy measurements were performed with a Labram HR800 UV confocal micro-Raman spectroscopy (Horiba, Japan). In the Raman measurement, the high-power helium-neon laser with a 632.8 nm wavelength was applied as the excitation light source and the scanning range was from 700 to 3800 cm^−1^. The specimen size for Raman measurement is 1 mm × 1 mm × 1.5 mm.

## 4. Results and Discussion

**Figure 2:**
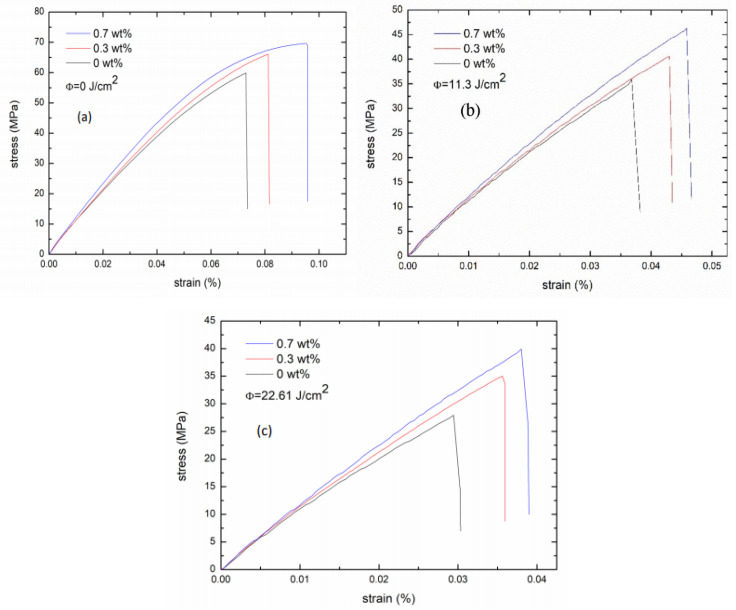
The stress–strain curves of PMMA/FGs composites with different UV doses: (**a**) 0 J/cm^2^, (**b**) 11.3 J/cm^2^, and (**c**) 22.6 J/cm^2^.

The tensile test was conducted to examine the effects of both FGs concentration and UV dose on the reinforcement in PMMA/FGs composites. Figure 2 shows the curves of stress versus strain for the PMMA/FGs composites with FGs of different concentrations irradiated with a UV dose of 11.3 J/cm^2^ where the fracture stress, Young’s modulus, and elongation at the break are tabulated in Table 1. The fracture stress, Young’s modulus, and elongation at the break increase with the increase of FGs concentration but decrease with the increase of UV dose at a fixed FGs concentration. Note that the values of fracture stress, Young’s modulus, and elongation at the break of pure PMMA are in the same order as those reported by Ali et al. [5]. Young’s modulus is increased by 14.6% for the PMMA/FGs composite of 0.7 wt% FGs concentration, which is less than a 33% increase for the PMMA/FGs composite of 0.01 wt% FGs concentration reported by Ramanathan et al. [19]. Ramanathan et al. used the solution-mixing method to obtain the specimen. They obtained a 5% increase in stiffness using Voigt’s upper bound calculation [19]. FGs have an affinity for PMMA. Adding carboxylic groups to FGs makes FGs chemically stable and provides a large specific surface area to effectively disperse the PMMA, as well as prevent agglomeration. According to Table 1, FGs enhance the mechanical strength of PMMA due to FGs hindering the chain motion of PMMA [37]. However, UV irradiation makes chain scission of PMMA and reduction in molecular weight, which leads to a decrease in the fracture stress, Young’s modulus, and elongation. Such a result can be explained by Raman spectroscopy.

**Figure 3:**
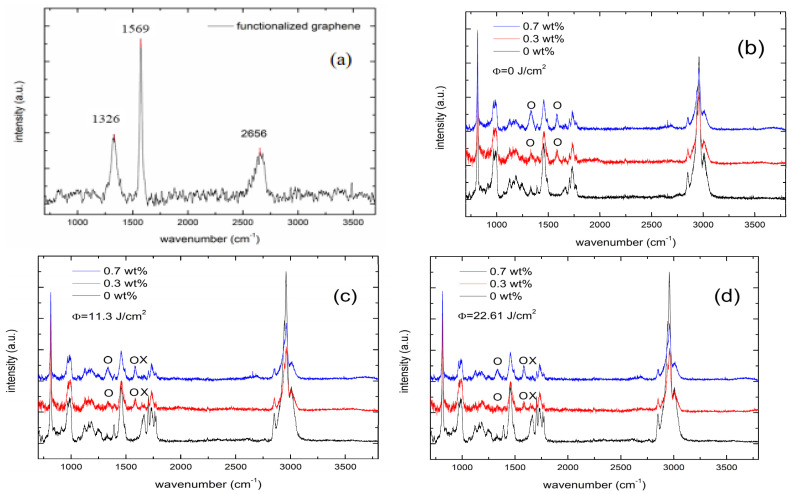
The Raman spectrum: (**a**) the functionalized graphene sheet, (**b**) PMMA/FGs composite without UV irradiation, (**c**) PMMA/FGs composite with a UV dose of 11.3 J/cm^2^, and (**d**) PMMA/FGs composite with a UV dose of 22.6 J/cm^2^. The symbols o and × represent D, G bands and extra peak induced by UV irradiation.

The Raman spectroscopy of the FG sheets was illustrated in Figure 3a. Two peaks located at 1326 cm^−1^ and 1569 cm^−1^ represent the D band and G band, respectively. D band is a disordered structure, induced by defects and unstable sp^3^ hybridization orbital C-C bond. G band is the stable sp^2^ C-C bond in the graphitic structure [38,39]. The Raman spectra of PMMA/FGs composite irradiated with UV doses of ϕ = 0, 11.3, and 22.6 J/cm^2^ are illustrated in Figure 3b–d, respectively. Two peaks are indicated by the hollow circle in Figure 3b–d which correspond to the positions of the D band and G band of the FGs. The ratio of the peak intensities of the D band to the G band, I_D_/I_G_, which is known as the defect degree, is used to investigate the chemical bonds of PMMA/FGs composites. The values of I_D_/I_G_ of FGs sheets and composites with different FGs concentrations are tabulated in Table 2. The I_D_/I_G_ values of composites are significantly larger than that of FGs. The strong interaction between the PMMA matrix and FGs increases the I_D_/I_G_ values because the FGs structure is modified by the AIBN and leads to a good dispersion in the matrix. The higher the concentration of FGs in the PMMA/FGs composite, the higher the I_D_/I_G_ value is. The strong interaction is important for graphene filler, graphene sheet dispersion, and matrix adhesion, which enhances the mechanical properties of the composites. Another piece of evidence for the uniform distribution of the FGs in PMMA/FGs composite is the low value of the standard deviation of the mechanical properties tabulated in Table 1. The Raman spectra of pure PMMA with different UV doses are shown in Figure S2 in Supplementary Information. A new small peak marked cross is located at 1647 cm^−1^ in Figure 3c,d and Figure S2 in Supplementary Information. This peak is attributed to the unsaturated C=C bonds induced by polymer chain scission [40,41]. The values of I_D_/I_G_ are observed to decrease with the increase of the UV dose. UV irradiation is capable of decreasing the defect concentration in FGs.

The plots of (*t*/*t*_0_ − 1)/*c* versus THF concentration for PMMA/FGs composite with different concentrations of FGs and UV doses are shown in Figure S3 in Supplementary Information. Huggins [42] proposed the following equation to obtain the intrinsic viscosity [*η*] of solute,

(7) $$\frac{1}{c}\left(\frac{\eta }{\eta_{0}}-1\right)=\frac{1}{c}\left(\frac{t}{t_{0}}-1\right)=\left[\eta \right]+k_{H}{\left[\eta \right]}^{2}c$$

where *c* is the mass concentration of solute, *t* and *t*_0_ are the flow times of solution and solvent, *η* and *η*_0_ are the viscosities of solution and solvent, and *k_H_* is a constant. Applying Equation (7) to Figure S3 in Supplementary Information, we obtain the intrinsic viscosities of PMMA/FGs composites with different concentrations of FGs and UV doses, which are tabulated in Table S1. The intrinsic viscosity increases with the increasing concentration of FGs, which indicates that the movement of the PMMA polymer chains in solvent becomes more difficult with the increase of FGs concentration. With the increasing UV exposure, the intrinsic viscosity decreases because UV exposure leads to polymer chain scission and reduces chain molecular weight.

Mark, Houwink, and Sakurada found the intrinsic viscosity and molecular weight, M, of polymer have the following relation, [43]

(8) $$[\eta ]\;=\;KM^{\alpha }$$

where K and *α* are constant and dependent on the polymer-solvent system. For the PMMA-THF system at 30 °C, K and *α* are 1.28 × 10^−4^ dL/g and 0.690, respectively [44]. Using Equation (8) and Table S1, one obtains the molecular weights of PMMA/FGs composites of different FGs concentrations irradiated with various UV doses and lists them in Table 3. The molecular weight decreases with the increasing UV dose, implying that UV irradiation makes chain scission and lowers the molecular weight. The molecular weight increases with the increasing FGs concentration; that is, adding FGs to the PMMA causes chain crosslinks and enhances the molecular weight.

**Figure 4:**
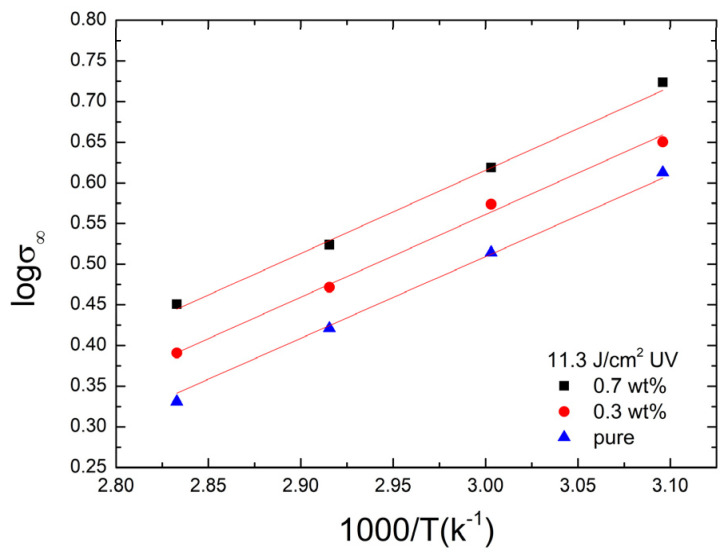
The plots of the logarithm of steady-state stress versus 1000/T for the PMMA/FGs composites irradiated with 11.3 J/cm^2^ UV dose.

For a given 0.5% strain and 11.3 J/cm^2^ UV dose, the variations of stress with time for PMMA/FGs composites with FGs of 0%, 0.3%, and 0.7% concentrations are shown in Figure 4a–c, respectively. Experimental data for composites without UV irradiation and with 22.62 J/cm^2^ doses are shown in Figures S4 and S5 in Supplementary Information, respectively, which has a similar trend to the composite irradiated with 11.3 J/cm^2^. The stress decreases exponentially with time and finally approaches a plateau. The higher the temperature, the sooner the plateau is reached. The steady-state stress decreases with the temperature but increases slightly with FGs concentration. Both initial stress and steady-state stress decrease with the increasing UV dose. The stress reduction results from the polymer chain scission induced by UV irradiation.

The steady-state stress, *σ*_∞_, is the residual stress in PMA/FGs composite to maintain the given strain (e.g., 0.5% strain). The plot of the log(*σ*_∞_) versus 1000/T for the different FGs concentrations of PMMA/FGs composites irradiated at 11.3 J/cm^2^ UV dose is exhibited in Figure 4. It is found that the data satisfies the van ’t Hoff equation,

(9) $$\sigma_{\infty }=A\;exp\left(-\frac{\Delta H_{v}}{RT}\right)$$

where *A* is the pre-exponential parameter, Δ*H_v_* is the enthalpy change of the deformation process, *R* gas constant, and *T* absolute temperature. Similar plots for the composites without UV irradiation and at 22.6 UV dose are shown in Figure S6a,b in Supplementary Information, respectively. The solid lines in Figure 4 and Figure S6a,b in Supplementary Information are obtained using Equation (9). The enthalpy changes of the deformation process for the PMMA/FGs composites under different UV irradiation doses are tabulated in Table 4. The negative sign of Δ*H_v_* indicates the deformation is an exothermal process [45]. The enthalpy changes are found to be roughly independent of the FGs concentration but decrease with the UV dose.

**Figure 5:**
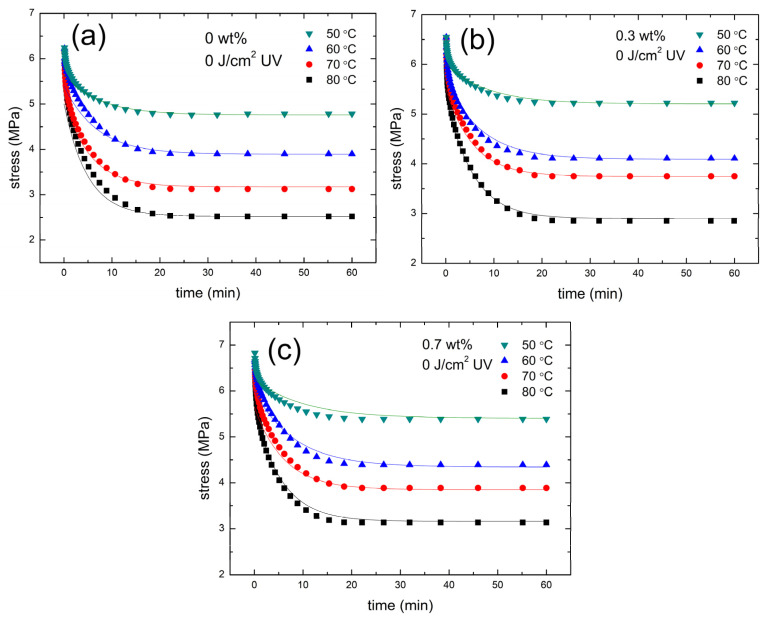
The stress–relaxation data at different temperatures for FGs/PMMA composite with (**a**) 0 wt% FGs, (**b**) 0.3 wt% FGs, and (**c**) 0.7 wt% FGs irradiated with 11.3 J/cm^2^ UV dose.

The Kelvin representation of SLSM (Equation (6) is applied to fit the stress–relaxation curves of PMMA/FGs composites. The solid lines in Figure 5, Figures S4 and S5 in Supplementary Information are obtained using Equation (6) with the parameters, E_1k_, E_2k_, and η_2k_ for doses 11.3, 0, and 22.6 J/cm^2^ being tabulated in Table 5, Tables S2 and S3 in Supplementary Information, respectively. It can be seen from Figure 5, Figures S4 and S5 in Supplementary Information that the experimental data are in good agreement with the theoretical prediction. The values of E_1k_, E_2k,_ and η_2k_ decrease with the increase of the temperature and increase with the increasing concentration of FGs. According to Equation (6) at the initial time, Young’s modulus of PMMA/FGs composite is equal to E_1k_. Comparing Table 1 with Table 5, Tables S1 and S2 in Supplementary Information, Young’s modulus of PMMA/FGs composite at room temperature is always greater than that at elevated temperature regardless of FGs concentration and UV irradiation.

The stress–relaxation data for the PMMA/FGs composites are shown in Figure 5, and Figures S4 and S5 in Supplementary Information can also be curve-fitted using the Maxwell representation of the SLSM model (See Equation (S6) in Supplementary Information). The fitting parameters of E_1m_, E_2m,_ η_2m,_ and β_s_ for the PMMA/FGs composites of different FGs concentrations with the UV dose of 11.3 J/cm^2^ are shown in Table 6. The fitting parameters of E_1m_, E_2m,_ η_2m_, and β_s_ for the PMMA/FGs composites with UV doses of 0 and 22.6 J/cm^2^ are listed in Tables S4 and S5 in the Supplementary Information. Comparing Table 5 and Table 6, we find the values of β_s_ and R^2^ are the same because both Kelvin and Maxwell representations are mathematically identical. Note that parameters of E_1m_, E_2m,_ and η_2m_ can be obtained from E_1k_, E_2k_, and η_2k_ using Equations (S7) and (S8) in Supplementary Information. It is found that E_1m_ decreases with the increasing temperature for all PMMA/FGs composites. However, the value of E_2m_ increases with the increase of temperature, which violates the concept of materials science. When the temperature increases, the atomic thermal vibrations increase, and this will cause the changes in lattice potential energy and curvature of the potential energy curve, so Young’s modulus will also change. Although the temperature dependence of Young’s modulus is very complicated, the trend of decreasing Young’s modulus with the increasing temperature is well known in polymeric materials [46,47,48].

Lagakos et al. [46] and Ferry [47] showed the S curves of log E versus 1/T for an amorphous polymer where E and T are Young’s modulus and temperature, respectively. They divided the S curve into three temperature regions. At low temperatures, the polymer is hard and brittle, and Young’s modulus decreases slowly with increasing temperature. The second one is the glass-rubber transition region where Young’s modulus decreases very rapidly by several orders of magnitude over a small temperature range. As the temperature is further increased, the polymer is in a rubber state, and Young’s modulus decreases slowly with temperature. In addition to the above three regions, Aklonis and MacKnight [48] claimed the fourth temperature region, where the temperature is greater than that in the third region for a linear polymer, and Young’s modulus decreases very rapidly with increasing temperature. The authors found that the plasticizer and molecular weight affect the modulus–temperature curve, but the trend of modulus as a decreasing temperature function does not change. It can be seen from Table 6 that E_2m_ increases with the increasing temperature for all PMMA/FGs composites and PMMA is an amorphous polymer, implying that the Maxwell representation of SLSM is not suitable to model the stress–relaxation behavior of PMMA/FGs composites.

**Figure 6:**
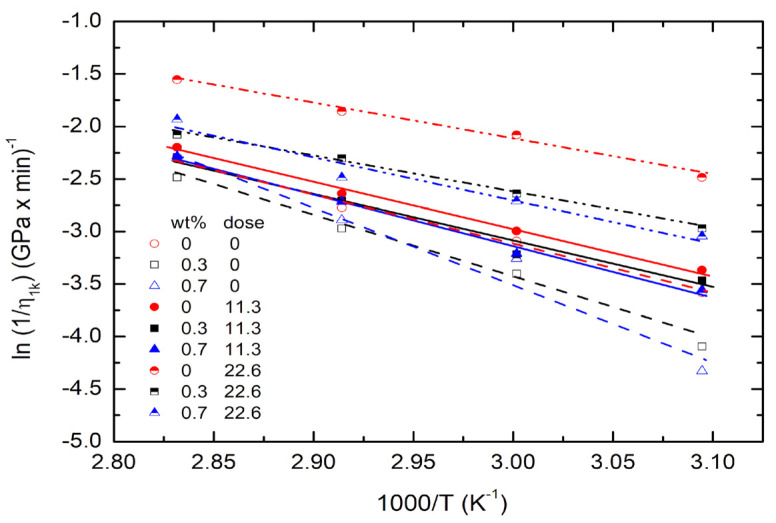
The plots of ln(1/$\eta_{1k})$ versus 1000/T for the composites of different concentrations of FGs and UV doses.

From Table 5 and Tables S2 and S3 in Supplementary Information, the plots of ln(1/*η*_2*k*_) versus 1000/T for PMMA/FGs composites with different concentrations of FGs and UV doses are shown in Figure 6. The solid lines are obtained using the Arrhenius equation,

(10) $$\frac{1}{\eta_{2k}}=\frac{1}{\eta_{0}}exp\left(-\frac{Q_{\eta }}{RT}\right)$$

where *Q_η_* and *η*_0_ are the activation energy and pre-exponential factor of the viscous deformation, *R* gas constant, and *T* the absolute temperature. The activation energy is calculated from the slope of each curve in Figure 6 and listed in Table 7. The activation energy increases with the increase of FGs concentration but decreases with the UV dose. This implies that viscous deformation occurs most easily when the pure PMMA is irradiated with a UV dose of 22.6 J/cm^2^. The viscous flow occurs most difficultly in the non-irradiated PMMA/FGs composites with 0.7 wt% FGs concentration. The chain scission results in the low molecular weight of the composites. It also enhances viscous deformation, and FGs inhibit the polymeric chain motion and reduce the viscous deformation.

## 5. Conclusions

The effect of UV irradiation on the stress relaxation behavior of the poly(methyl methacrylate)/functionalized graphene (PMMA/FGs) composite is investigated. The fracture stress, Young’s modulus, and elongation at break increase with the increase of the FGs concentration, but they have the opposite trend to UV irradiation. The molecular weight of the PMMA/FGs composite increases with the increasing concentration of FGs but decreases with UV dose. The FGs hinder the polymer chain flow and cause the chain crosslinks so that the composite with large FGs concentration has high mechanical strength. UV irradiation makes chain scission so that the PMMA/FGs composite is irradiated with a great UV dose, which chains move easily, and has low mechanical strength. The Kelvin representation of the standard linear solid model (SLSM) is used to simulate the stress–relaxation data of the PMMA/FGs composite. The reciprocal of viscosity follows the Arrhenius equation, and its activation energy increases with increasing concentration of FGs and decreasing UV dose. Although the Maxwell representation of SLSM can fit the stress–relaxation data of the PMMA/FGs composite and its Young’s modulus increases with the increasing temperature, which violates the concept of Young’s modulus as a decreasing temperature function for amorphous polymers. Therefore, the Maxwell representation of SLSM is not suitable to simulate the PMMA/FGs composite. The steady-state stress satisfies the van ’t Hoff equation. The enthalpy change of the stress–relaxation process has a negative value, implying that stress relaxation is an exothermal process. The enthalpy change is independent of FGs concentration and decreases with the increasing UV dose.

## Figures and Tables

**Table 1:** The summarized data and their standard deviations of fracture stress, Young’s modulus, and elongation at break of the PMMA/FGs composites with different UV doses.

FGs (wt%)	Fracture Stress (MPa)			Young’s Modulus (GPa)			Elongation at Break (%)		
	0 (J/cm^2^)	11.3 (J/cm^2^)	22.61 (J/cm^2^)	0 (J/cm^2^)	11.3 (J/cm^2^)	22.61 (J/cm^2^)	0 (J/cm^2^)	11.3 (J/cm^2^)	22.61 (J/cm^2^)
0	60.98 ± 1.43	35.97 ± 3.27	28.11 ± 3.43	1.23 ± 0.02	1.16 ± 0.04	1.08 ± 0.02	7.39 ± 0.34	3.57 ± 0.64	2.97 ± 0.21
0.3	65.94 ± 1.42	40.53 ± 5.31	36.05 ± 3.18	1.30 ± 0.04	1.29 ± 0.03	1.21 ± 0.04	8.02 ± 0.46	4.21 ± 0.33	3.66 ± 0.54
0.7	70.53 ± 1.05	48.76 ± 2.40	39.08 ± 5.48	1.41 ± 0.02	1.31 ± 0.03	1.25 ± 0.04	9.53 ± 0.57	4.55 ± 0.42	4.06 ± 0.70

**Table 2:** The I_D_/I_G_ ratio of FGs and PMMA/FGs composites with different UV doses.

FGs (wt%)	UV Dose (J/cm^2^)	I_D_	I_G_	I_D_/I_G_
100	0	18.22	46.46	0.39
0.3	0	44.68	48.05	0.93
	11.3	36.18	45.96	0.79
	22.61	25.65	37.48	0.68
0.7	0	62.48	52.15	1.20
	11.3	42.04	44.83	0.94
	22.61	39.15	50.81	0.77

**Table 3:** The molecular weights of PMMA and PMMA/FGs composites with different FGs concentrations and the various UV doses.

FGs	Dose		
	0 J/cm^2^	11.3 J/cm^2^	22.61 J/cm^2^
0 wt%	1.32 × 10^5^	9.61 × 10^4^	5.04 × 10^4^
0.3 wt%	1.51 × 10^5^	1.09 × 10^5^	8.48 × 10^4^
0.7 wt%	1.62 × 10^5^	1.23 × 10^5^	8.86 × 10^4^

**Table 4:** The enthalpy changes of the deformation process for PMMA/FGs composites irradiated with different UV doses.

FGs		Dose	
	0 J/cm^2^	11.3 J/cm^2^	22.6 J/cm^2^
0 wt%	−21.08 kJ/mol	−19.14 kJ/mol	−18.97 kJ/mol
0.3 wt%	−21.20 kJ/mol	−19.49 kJ/mol	−18.32 kJ/mol
0.7 wt%	−21.36 kJ/mol	−19.56 kJ/mol	−18.11 kJ/mol

**Table 5:** The parameters fitted the stress–relaxation data at different temperatures using the Kelvin representation of SLSM for PMMA/FGs composites with an irradiated dose of 11.3 J/cm^2.^

Temperature	80 °C			70 °C			60 °C			50 °C		
FGs (wt%)	0	0.3	0.7	0	0.3	0.7	0	0.3	0.7	0	0.3	0.7
E_1k_ (GPa)	0.9	0.95	0.95	0.95	1.02	1.02	1.02	1.07	1.07	1.06	1.1	1.1
E_2k_ (GPa)	0.7	0.84	0.84	1.35	1.47	1.47	2.01	2.33	2.33	3.	3.3	3.3
η_2k_ (GPa × min)	9.	10.	9.8	14	15.	15.1	20.	25	24.8	29	32	35
β_s_ (min)	0.178	0.179	0.212	0.164	0.166	0.164	0.152	0.136	0.128	0.140	0.138	0.119
R^2^	0.990	0.994	0.995	0.989	0.986	0.986	0.992	0.986	0.988	0.988	0.982	0.991

**Table 6:** The parameters E_1m_, E_2m_, η_2m,_ and β_s_ at different temperatures for PMMA/FGs composites with an irradiated dose of 11.3 J/cm^2.^

Temperature	80 °C			70 °C			60 °C			50 °C		
FGs (%)	0	0.3	0.7	0	0.3	0.7	0	0.3	0.7	0	0.3	0.7
E_1m_ (GPa)	0.394	0.446	0.475	0.558	0.602	0.675	0.677	0.733	0.826	0.783	0.825	0.899
E_2m_ (GPa)	0.506	0.504	0.585	0.392	0.418	0.415	0.343	0.337	0.314	0.277	0.275	0.261
η_2m_ (GPa × min)	2.848	2.817	2.76	2.388	2.517	2.533	2.266	2.476	2.46	1.977	2.00	2.19
β_s_ (min)	0.178	0.179	0.212	0.164	0.166	0.164	0.152	0.136	0.128	0.140	0.138	0.119
R^2^	0.990	0.994	0.995	0.989	0.986	0.986	0.992	0.986	0.988	0.988	0.982	0.991

**Table 7:** The activation energies *Q_η_* and confidential intervals R^2^ of the viscous behavior in the PMMA/FGs composites with different FGs concentrations and UV doses.

Dose FGs	0 J/cm^2^		11.3 J/cm^2^		22.6 J/cm^2^	
	*Q_η_* (kJ/mol)	R^2^	*Q_η_* (kJ/mol)	R^2^	*Q_η_* (kJ/mol)	R^2^
0 wt%	39.47	0.994	36.63	0.995	28.66	0.990
0.3 wt%	49.99	0.993	37.84	0.979	28.52	0.996
0.7 wt%	61.39	0.962	40.87	0.992	33.68	0.960

## Data Availability

The data presented in this study are available on request from the corresponding author.

## Supplementary Materials

The following supporting information can be downloaded at: https://www.mdpi.com/article/10.3390/polym14194192/s1, Figure S1: Schematic of Maxwell representation of SLSM, Figure S2: Raman spectra, Figure S3: Intrinsic viscosity, Figures S4 and S5: Stress relaxation data, Figure S6: Van’t Hoff plot, Table S1: Intrinsic viscosity values, Tables S2 and S3: The parameters of Kelvin representation for UV doses of 0 and 22.06 J/cm^2^, Tables S4 and S5: The parameters of Maxwell representation for UV doses of 0 and 22.06 J/cm^2^
