# Supplementary material for: Stress Relaxation Behavior of Poly(Methyl Methacrylate)/Graphene Composites: Ultraviolet Irradiation
Source: Polymers (Basel). 2022 Oct 6;14(19):4192. doi: 10.3390/polym14194192 (PMC9573155; doi:10.3390/polym14194192)
Supplement: Supplementary file 1 [file polymers-14-04192-s001.zip › polymers-1944152-supplementary.pdf]

Stress relaxation behavior of polymethylmethacrylate/graphene composites: ultraviolet irradiation

Yu-cheng Ju[1], Donyau Chiang[2], Ming-Yen Tsai[1], Hao Ouyang[1], Sanboh Lee*[,1]

1) Department of Material Science and Engineering, National Tsing Hua University, Hsinchu 300, Taiwan

2) Taiwan Instrument Research Institute, National Applied Research Laboratories, Hsinchu 300, Taiwan

1. Standard linear solid model: Maxwell representation

The Maxwell representation of standard linear solid model (SLSM), as shown in Fig. S1, is also used to model the stress relaxation behavior of polymeric composite. For the Maxwell representation, the spring 1m is connected in parallel with the Maxwell element, which consists of a spring 2m and a dashpot 2m connected in series. The mathematical manipulation of Maxwell representation of SLSM is similar to that of Kelvin representation. The total stress consists of stress in spring 1m and stress in Maxwell element.

$$\sigma = \sigma_1 + \sigma_2 = E_{1m}\varepsilon_{1s} + E_{2m}\varepsilon_{2a} = E_{1m}\varepsilon_{1s} + \eta_{2m}\frac{d\varepsilon_{2b}}{dt} \quad \text{(S1)}$$

The applied stress in spring 2m is the same as that in dashpot 2m in Maxwell element.

$$E_{2m}\varepsilon_{2a} = \eta_{2m}\frac{d\varepsilon_{2b}}{dt} \quad \text{(S2)}$$

The strain of spring 1m is equal to the sum of both strains for spring 2m and dashpot 2m.

$$\varepsilon_{1s} = \varepsilon_{2a} + \varepsilon_{2b} \quad \text{(S3)}$$

Substituting Eq. (S3) into Eq. (S2) yields

$$E_{2m}\varepsilon_{2a} = \eta_{2m}\frac{d(\varepsilon_{1s}-\varepsilon_{2a})}{dt} \quad \text{(S4)}$$

Integrating both sides of Eq. (S4) with the initial condition of $\varepsilon_{1s} = \varepsilon_{2a}$ yields Eq. (S5) as,

$$\varepsilon_{2a} = \varepsilon_{1s}exp\,(-\frac{E_{2m}t}{\eta_{2m}}) \quad \text{(S5)}$$

Substituting Eq. (S5) into Eq. (S1), one obtains the stress-time relation of Maxwell representation of SLSM for stress relaxation as

$$\sigma(t) = E_{1m}\varepsilon_{1s} + E_{2m}\varepsilon_{1s}exp\left(-\frac{E_{2m}t}{\eta_{2m}}\right) = E_{1m}\varepsilon_{1s} + E_{2m}\varepsilon_{1s}exp(-\beta_s t) \quad \text{(S6)}$$

The term β_s is defined as relaxation rate, which is the same value in Eq. (6). It is found that Kelvin and Maxwell representations of SLSM are mathematically identical if their parameters satisfy Eqs. (S7) and (S8),

$$E_{1m} = \frac{E_{1k}E_{2k}}{E_{1k}+E_{2k}} \; and \; E_{2m} = \frac{{E_{1k}}^2}{E_{1k}+E_{2k}} \tag{S7}$$

$$\eta_{2m} = (\frac{E_{1k}}{E_{1k}+E_{2k}})^2\eta_{2k} \tag{S8}$$

Note that Eqs. (S7) and (S8) are obtained by comparing constant, pre-exponential, and exponent terms of Eqs. (6) and (S6).

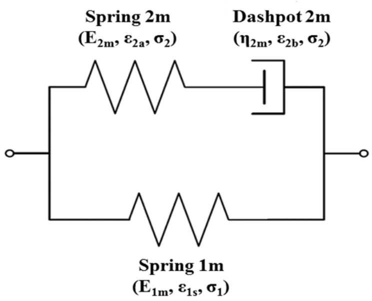

Figure S1. Schematics of standard linear solid model for stress relaxation: Maxwell representation.

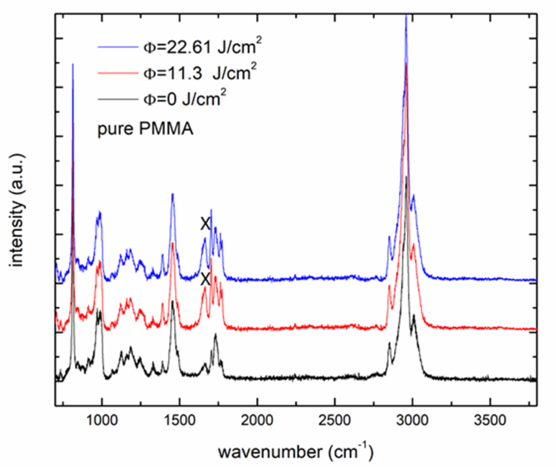

Figure S2. Raman spectra of the PMMA irradiated with different UV doses.

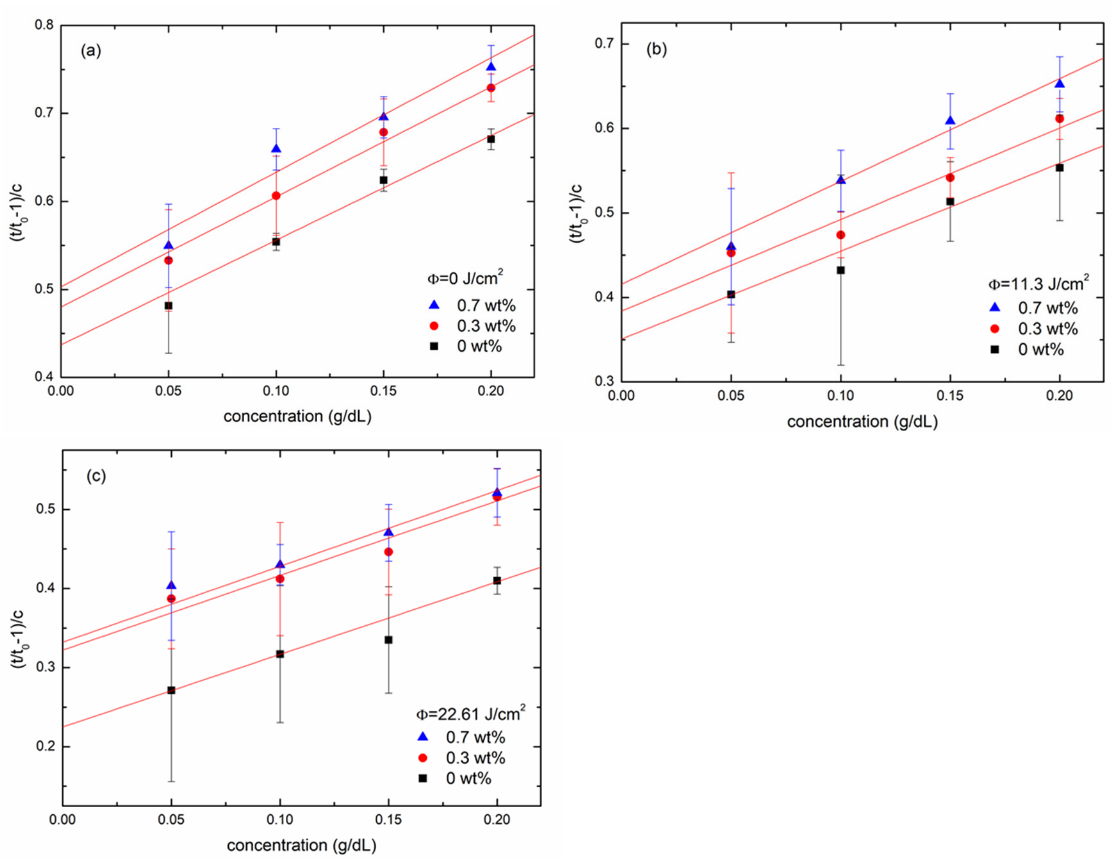

Figure S3. The plots of $(t/t_0\text{-}1)/c$ versus THF concentration for PMMA/FGs composites irradiated with different doses: (a) $\phi = 0$ J/cm^2, (b) $\phi = 11.3$ J/cm^2, and (c) $\phi = 22.61$ J/cm^2.

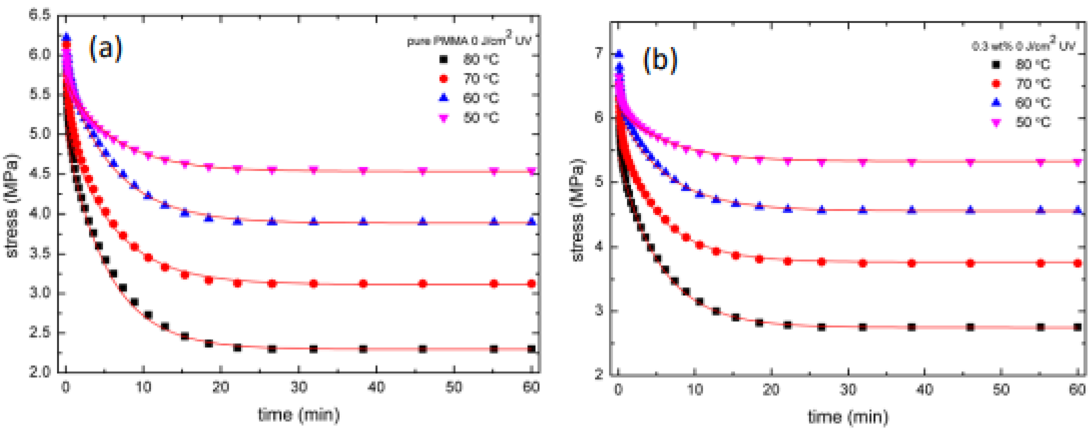

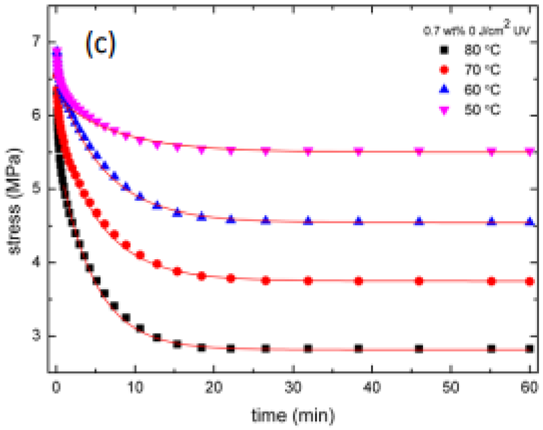

Figure S4. The plots of stress versus time for (a) pure PMMA, (b) 0.3 wt% FGs/PMMA composite, and (c) 0.7 wt% FGs/PMMA composite at different temperatures without UV irradiation.

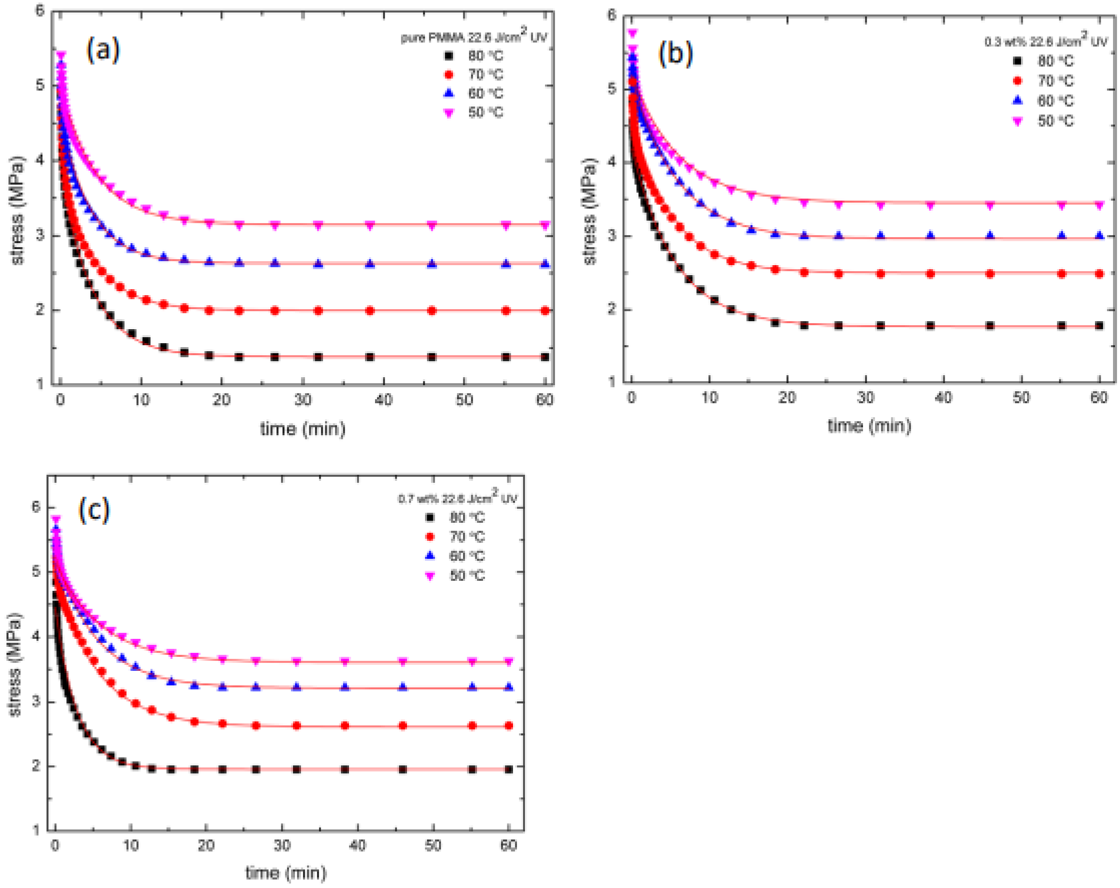

Figure S5. The plots of stress versus time at different temperatures for (a) pure PMMA, (b) 0.3 wt% PMMA/FGs composite, and (c) 0.7 wt% PMMA/FGs composite with UV dose of 22.6 J/cm^2.

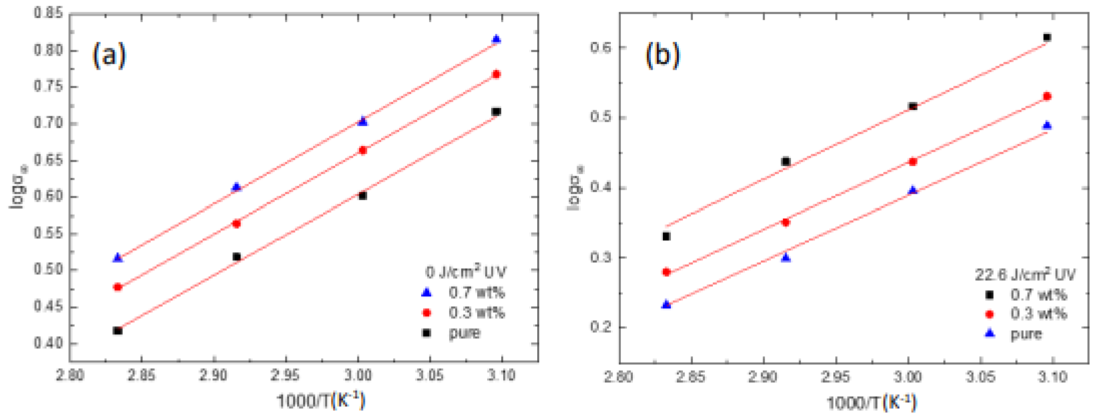

Figure S6. The plot of logσ∞ versus 1000/T for PMMA/FGs composites with various concentrations of FGs with different UV doses: (a) ϕ=0 and (b) ϕ= 22.6 J/cm^2.

Table S1. The intrinsic viscosity values in the unit of dL/g for PMMA and PMMA/FGs composites with different FGs concentrations and the various UV doses.

	Dose		
FGs	0 J/cm^2	11.3 J/cm^2	22.61 J/cm^2
0 wt%	0.437	0.351	0.225
0.3 wt%	0.480	0.384	0.322
0.7 wt%	0.503	0.416	0.332

Table S2. The parameters, E_{1k}, E_{2k}, and η_{2k} were used to fit the stress relaxation curves at different temperatures for non-irradiated PMMA/FGs composites with different concentrations of FGs.

Temperature	80°C			70°C			60°C			50°C		
FGs content (%)	0	0.3	0.7	0	0.3	0.7	0	0.3	0.7	0	0.3	0.7
E_{1k} (GPa)	1.0	1.08	1.12	1.02	1.12	1.18	1.15	1.22	1.3	1.13	1.23	1.25
E_{2k} (GPa)	0.85	1.12	1.13	1.6	2.3	2.06	2.4	3.6	3.03	4.6	8.0	9.2
η_{2k}(GPa×min)	10	12	10	16	19.5	18	22	30.	26	36	60.	75.8
β_s(min)	0.185	0.183	0.225	0.164	0.175	0.180	0.161	0.161	0.167	0.159	0.154	0.138
R^2	0.988	0.992	0.993	0.991	0.987	0.986	0.985	0.982	0.988	0.978	0.965	0.984

Table S3. The parameters, E_{1k}, E_{2k}, and η_{2k} were used to fit the stress relaxation curves at different temperatures for PMMA/FGs composites with different concentrations of FGs and UV dose of 22.6 J/cm^2.

Temperature	80°C			70°C			60°C			50°C		
FGs content (%)	0	0.3	0.7	0	0.3	0.7	0	0.3	0.7	0	0.3	0.7
E_{1k} (GPa)	0.77	0.84	0.89	0.8	0.92	0.98	0.92	1.03	1.05	1.	1.06	1.03
E_{2k} (GPa)	0.43	0.61	0.7	0.8	1.05	1.12	1.23	1.4	1.66	1.7	2.15	2.4
η_{2k}(GPa×min)	4.73	8.	6.9	6.4	10	12	8.	14	15.03	12	19.45	21
β_s(min)	0.254	0.181	0.230	0.250	0.197	0.175	0.269	0.174	0.180	0.225	0.165	0.163
R^2	0.989	0.987	0.989	0.991	0.992	0.990	0.990	0.984	0.987	0.985	0.986	0.985

Table S4. The parameters, E_{1m}, E_{2m}, and η_{2m} were used to fit stress relaxation curves at different temperatures for the non-irradiated PMMA/FGs composites with FGs of different concentrations

Temperature	80°C			70°C			60°C			50°C		
FGs content (%)	0	0.3	0.7	0	0.3	0.7	0	0.3	0.7	0	0.3	0.7
E_{1m} (GPa)	0.459	0.55	0.56	0.623	0.75	0.75	0.777	0.91	0.91	0.907	1.07	1.10
E_{2m} (GPa)	0.541	0.53	0.56	0.397	0.37	0.43	0.373	0.31	0.39	0.223	0.16	0.15
η_{2m}(GPa×min)	2.922	2.89	2.48	2.425	2.09	2.39	2.309	1.92	2.34	1.40	1.07	1.15
β_s(min)	0.185	0.183	0.225	0.164	0.175	0.18	0.161	0.16	0.167	0.159	0.154	0.131
R^2	0.988	0.992	0.993	0.991	0.987	0.986	0.985	0.982	0.988	0.978	0.965	0.984

Table S5. The parameters, E_{1m}, E_{2m}, and η_{2m} were used to fit stress relaxation at different temperatures for the PMMA/FGs composites with FGs of different concentrations and UV dose of 22.6 J/cm^2

Temperature	80°C			70°C			60°C			50°C		
FGs content (%)	0	0.3	0.7	0	0.3	0.7	0	0.3	0.7	0	0.3	0.7
E_{1m} (GPa)	0.276	0.35	0.39	0.40	0.49	0.52	0.526	0.59	0.64	0.63	0.71	0.72
E_{2m} (GPa)	0.494	0.49	0.50	0.40	0.43	0.46	0.394	0.44	0.41	0.37	0.35	0.31
η_{2m}(GPa×min)	1.948	2.68	2.16	1.60	2.18	2.61	1.465	2.52	2.26	1.65	2.121	1.894
β_s(min)	0.254	0.181	0.230	0.25	0.197	0.175	0.269	0.174	0.18	0.225	0.165	0.163
R^2	0.989	0.987	0.989	0.991	0.992	0.990	0.990	0.984	0.987	0.985	0.986	0.985
